# Framework for a Community Health Observing System for the Gulf of Mexico Region: Preparing for Future Disasters

**DOI:** 10.3389/fpubh.2020.578463

**Published:** 2020-10-15

**Authors:** Paul Sandifer, Landon Knapp, Maureen Lichtveld, Ruth Manley, David Abramson, Rex Caffey, David Cochran, Tracy Collier, Kristie Ebi, Lawrence Engel, John Farrington, Melissa Finucane, Christine Hale, David Halpern, Emily Harville, Leslie Hart, Yulin Hswen, Barbara Kirkpatrick, Bruce McEwen, Glenn Morris, Raymond Orbach, Lawrence Palinkas, Melissa Partyka, Dwayne Porter, Aric A. Prather, Teresa Rowles, Geoffrey Scott, Teresa Seeman, Helena Solo-Gabriele, Erik Svendsen, Terry Tincher, Juli Trtanj, Ann Hayward Walker, Rachel Yehuda, Fuyuen Yip, David Yoskowitz, Burton Singer

**Affiliations:** ^1^Center for Coastal Environmental and Human Health, College of Charleston, Charleston, SC, United States; ^2^School of Public Health and Tropical Medicine, Tulane University, New Orleans, LA, United States; ^3^Master's Program in Environmental and Sustainability Studies, College of Charleston, Charleston, SC, United States; ^4^School of Global Public Health, New York University, New York, NY, United States; ^5^Department of Agricultural Economics and Agribusiness, Louisiana State University, Baton Rouge, LA, United States; ^6^School of Biological, Environmental, and Earth Sciences, University of Southern Mississippi, Hattiesburg, MS, United States; ^7^Huxley College of the Environment, Western Washington University, Bellingham, WA, United States; ^8^Department of Global Health, University of Washington, Seattle, WA, United States; ^9^Gillings School of Global Public Health, University of North Carolina, Chapel Hill, NC, United States; ^10^Woods Hole Oceanographic Institution, Woods Hole, MA, United States; ^11^Rand Corporation, Pittsburg, PA, United States; ^12^Harte Research Institute, Texas A&M University-Corpus Christi, Corpus Christi, TX, United States; ^13^Scripps Institution of Oceanography, La Jolla, CA, United States; ^14^Department of Health and Human Performance, College of Charleston, Charleston, SC, United States; ^15^Computational Epidemiology Lab, Harvard Medical School, Boston, MA, United States; ^16^Department of Epidemiology and Biostatistics, Bakar Computational Health Sciences Institute, University of California, San Francisco, San Francisco, CA, United States; ^17^Gulf of Mexico Coastal Ocean Observing System, Texas A&M University, College Station TX, United States; ^18^Laboratory of Neuroendocrinology, Rockefeller University, New York, NY, United States; ^19^Emerging Pathogens Institute, University of Florida, Gainesville, FL, United States; ^20^Department of Mechanical Engineering, University of Texas, Austin, TX, United States; ^21^Suzanne Dworak-Peck School of Social Work, University of Southern California, Los Angeles, CA, United States; ^22^Mississippi-Alabama Sea Grant Consortium, Mobile, AL, United States; ^23^Arnold School of Public Health, University of South Carolina, Columbia, SC, United States; ^24^Department of Psychiatry and Behavioral Sciences, University of California, San Francisco, San Francisco, CA, United States; ^25^National Marine Fisheries Service, National Oceanic and Atmospheric Administration, Silver Spring, MD, United States; ^26^David Geffen School of Medicine, University of California, Los Angeles, Los Angeles, CA, United States; ^27^Department of Civil, Architectural, and Environmental Engineering, University of Miami, Coral Gables, FL, United States; ^28^Division of Environmental Health Science and Practice, National Center for Environmental Health, Centers for Disease Control and Prevention, Atlanta, GA, United States; ^29^Office of Oceanic and Atmospheric Research, National Oceanic and Atmospheric Administration, Silver Spring, MD, United States; ^30^SEA Consulting Group, Cape Charles, VA, United States; ^31^Icahn School of Medicine at Mount Sinai, Bronx, NY, United States

**Keywords:** health observing system, disasters, Gulf of Mexico, cohort studies, stress, COVID-19, allostatic load, health surveillance

## Abstract

The Gulf of Mexico (GoM) region is prone to disasters, including recurrent oil spills, hurricanes, floods, industrial accidents, harmful algal blooms, and the current COVID-19 pandemic. The GoM and other regions of the U.S. lack sufficient baseline health information to identify, attribute, mitigate, and facilitate prevention of major health effects of disasters. Developing capacity to assess adverse human health consequences of future disasters requires establishment of a comprehensive, sustained community health observing system, similar to the extensive and well-established environmental observing systems. We propose a system that combines six levels of health data domains, beginning with three existing, national surveys and studies plus three new nested, longitudinal cohort studies. The latter are the unique and most important parts of the system and are focused on the coastal regions of the five GoM States. A statistically representative sample of participants is proposed for the new cohort studies, stratified to ensure proportional inclusion of urban and rural populations and with additional recruitment as necessary to enroll participants from particularly vulnerable or under-represented groups. Secondary data sources such as syndromic surveillance systems, electronic health records, national community surveys, environmental exposure databases, social media, and remote sensing will inform and augment the collection of primary data. Primary data sources will include participant-provided information via questionnaires, clinical measures of mental and physical health, acquisition of biological specimens, and wearable health monitoring devices. A suite of biomarkers may be derived from biological specimens for use in health assessments, including calculation of allostatic load, a measure of cumulative stress. The framework also addresses data management and sharing, participant retention, and system governance. The observing system is designed to continue indefinitely to ensure that essential pre-, during-, and post-disaster health data are collected and maintained. It could also provide a model/vehicle for effective health observation related to infectious disease pandemics such as COVID-19. To our knowledge, there is no comprehensive, disaster-focused health observing system such as the one proposed here currently in existence or planned elsewhere. Significant strengths of the GoM Community Health Observing System (CHOS) are its longitudinal cohorts and ability to adapt rapidly as needs arise and new technologies develop.

## Introduction

The Gulf of Mexico (GoM) region has experienced frequent disasters, including major and minor hurricanes and tropical storms as well as the massive Deepwater Horizon oil spill (DWH) (1). In addition to those disaster events emanating from Gulf waters, there are 872 “highly hazardous chemical facilities” in operation within 80 km of the GoM coast (2), and at least three major chemical plant explosions occurred in 2019 alone. The potential for chemical exposure is further exacerbated by numerous oil spills and seeps, some of significant magnitude and duration (3), and abandoned hazardous waste sites (4). Also, the GoM has experienced frequent and sustained periods of harmful algal blooms (HABs), with potential for human exposure to HAB toxins via surface water, seafood, and air (5). The GoM will likely experience continued frequent environmental and technological disasters, especially in this era of climate change and reduced environmental regulation (6–8). Most recently, the GoM States have been challenged by the COVID-19 pandemic, which differs from all of the others in its long duration and global geographical coverage.

Mental health impacts are a dominant effect of disasters (9), and disaster-related elevated stress may cause or exacerbate mental and physical disorders (10, 11). Adverse physical health effects of disasters beyond immediate and near-term injuries are less well-studied than mental outcomes. However, a variety of physical disorders have been linked to disaster experiences, including cardiovascular disease (CVD); asthma and other respiratory problems; digestive and intestinal complaints; eye, skin, and throat irritation; elevated blood pressure and heart rate; and some infectious and chronic diseases (12–15).

Repeated exposure to disaster events can amplify mental, physical, and community health effects. Children and adolescents may be especially vulnerable to impacts of multiple traumas (16–20). Pregnant women and mothers with young children may also be particularly vulnerable to disaster effects. Not surprisingly, the U.S. Department of Health and Human Services emphasizes that interventions should include elements specifically designed for children and adolescents (21). Elderly people, especially those with chronic conditions, are also of special concern because of potential for loss or interruption of health care and medications, inability to evacuate or move for treatment, heightened vulnerability during transport and dislocation, and loss of social contact and care mechanisms (15, 22–24). Now added to the cumulative trauma effects of recent hurricanes from Katrina through Michael as well as the DWH on the GoM population is the COVID-19 pandemic, with no clear end in sight. The recent social distancing and “stay at home” interventions of COVID-19 have resulted in stressful intra-household dynamics, psychological issues, and major economic concerns (25–28).

Although the region has a history of repeated, major environmental disasters, the GoM lacks a significant, continuing baseline of human health information that would enable the identification, comparison, and mitigation of health outcomes following disasters. This gap was highlighted by the National Commission on the BP Deepwater Horizon Oil Spill and Offshore Drilling (29), which specifically called out the need for a “public health protocol requiring the collection of adequate baseline data and long-term monitoring,” as well as by others (30). Health data should be collected over a period long after a given event has concluded in order to understand the full magnitude of effects and better prepare to deal with impacts of future disasters. Based on previous work (14, 31–33), studies spanning multiple decades are warranted to gauge long-term and transgenerational effects.

Major disasters like Hurricane Katrina, the DWH, and COVID-19 underscore the necessity for establishing long-term human health observations to improve disaster preparation. A sustained health observing system is needed in the GoM, analogous to observing systems that concentrate on high-intensity, relatively low-frequency-of-occurrence extreme weather events such as hurricanes (34–36). To be able to provide evidence to inform prevention, preparedness, response, and recovery actions, an effective disaster-focused health observing system must have capacity to collect relevant health data from cumulative impacts of sequential events and consequences of slower-moving, potentially devastating occurrences such as persistent environmental health threats, historical burdens of health disparities, chronic chemical contamination, drought, and climate change. Parker et al. (37) concluded that such planned pre–post studies for disasters are “virtually non-existent” and that “well-designed surveys with large probability-based samples and longitudinal assessment across the life-cycle of a disaster and across multiple disasters” are required. The current COVID-19 pandemic underscores the necessity to develop and implement much more robust health surveillance systems in the US and globally (38).

We propose the creation of a Gulf of Mexico Community Health Observing System (GoM CHOS) focused on effects of disasters on the health and well-being of people and their communities, which would operate continuously, producing pre-, during-, and post-disaster information. This will require the integration of available human health information with new and innovative approaches for measuring adverse health effects and community vulnerability. The primary objectives of the proposed GoM CHOS are to establish an ongoing system for the collection, analysis, and interpretation of a broad range of mental, physical, and community health data from a representative sample of GoM residents. The proposed system will (1) provide a continuous baseline of information against which to assess health impacts of future environmental, technological, and other disasters, individually and cumulatively; (2) implement an intensive data collection period in the immediate and near-term aftermath of disasters; and (3) substantially enhance clinical databases, thereby providing information for hypothesis generation and improving clinical and public health research and practice. The framework presented here includes observing system design, proposed sampling area and population sampling approaches, participant recruitment and retention, collection and assimilation of primary and secondary data, data management, and system governance. To the best of our knowledge, this is the first proposal for a disaster-specific health observing system in any location, and the system as outlined should be adaptable to many geographies and kinds of disasters.

## Methods

Recognizing that no framework or platform existed for a sustained community health observing system focused on disaster impacts, the Research Board of the Gulf of Mexico Research Initiative (GoMRI) commissioned the present work. The project encompassed efforts to (1) identify a set of essential data elements and (2) determine the potential for organizing available data, ongoing health information collection efforts, new health observing capacity, and technology into a comprehensive community health observing system.

The project was led by two Principal Investigators (P. Sandifer and B. Singer) and a Steering Committee of internationally recognized experts. Two expert workshops were convened to explore options for such an observing system, other subject matter experts were consulted, and a large body of literature and ongoing health surveys and studies was reviewed. Expert workshop 1 focused on the overall concept of a health observing system for the GoM region, while workshop 2 focused on the potential to operationalize the allostatic load concept of cumulative stress impacts on health for application in long-term health studies. Design of the proposed GoM CHOS was also informed by the highly successful environmental observing systems in place at regional, national, and global scales [e.g., GEO www.earthobservations.org; https://www.earthobservations.org/geoss.php NOAA's National Weather Service (www.weather.gov)], IOOS (ioos.noaa.gov) (39–42), and which provide information on atmospheric, oceanic, climate, weather, and biological conditions critical to life and livelihoods. Further details are provided in (1), which serves as a repository for information generated by the project.

## Results

### GoM CHOS Framework

**Figure 1:**
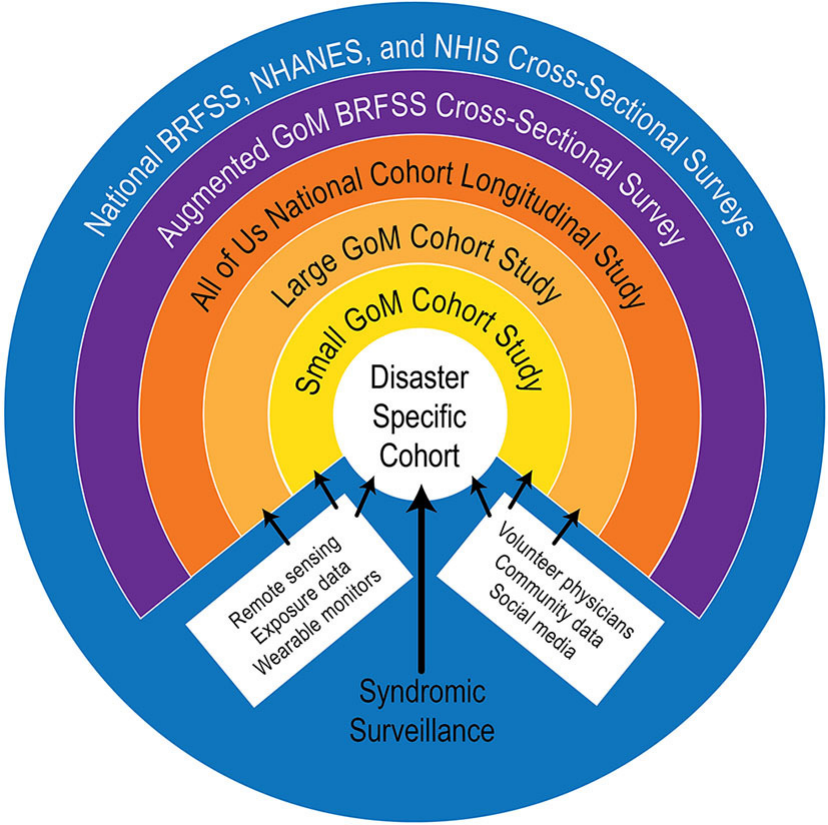
Diagram of a conceptual framework for a Gulf of Mexico Community Health Observing System (GoM CHOS). The All of us study (green ring) is under development by the National Institutes of Health and is expected to provide useful comparison data, as well as other materials, if it becomes fully operational as planned.

Based on essential requirements, guiding principles, and core values identified during our first workshop (1), an observing system framework was developed consisting of six levels of data domains, illustrated as concentric circles (Figure 1). These data domains encompass existing, large-scale surveys and studies as well as three new GoM-specific cohort studies.

The outer blue ring is the observing system's “backbone” of national surveys and data domains, encompassing the National Health and Nutrition Examination Survey (NHANES) (https://www.cdc.gov/nchs/nhanes/index.htm), the Behavioral Risk Factor Surveillance Survey (BRFSS) (https://www.cdc.gov/brfss/index.html), and the National Health Interview Survey (NHIS) (https://www.cdc.gov/nchs/nhis/index.htm). These are ongoing cross-sectional studies conducted by public agencies, and the data are available for research with restrictions to protect privacy. In addition to other data, they collect information on obesity, CVD, asthma, diabetes, and some other disorders that may increase individuals' vulnerability to disaster impacts and may be useful for comparison with data derived from the proposed cohort studies. BRFSS data are useful at state and periodically at county levels, while NHANES and NHIS data generally cannot be disaggregated for state-level comparisons. Collectively, these surveys provide a wealth of demographic, general health status, socioeconomic, and behavioral information [see (43) and websites for each survey].

The purple ring is a proposed augmented GoM BRFSS, in which additional questions pertinent to the GoM CHOS could be developed and asked annually by State Health Departments in the five GoM States, similar to the Gulf States Population Survey (GSPS) (44) conducted following the DWH event. The proposed new effort would enhance the richness of BRFSS collections, both spatially and in the form of disaster-relevant information. Implementation of an augmented BRFSS would require agreement by and additional funding for each of the GoM State Health Departments.

The orange ring is the NIH All of Us longitudinal cohort study, which has a target enrollment of 1 million adult participants that reflect the nation's diversity, including groups historically under-represented in biomedical research (45, 46). It began enrolling participants in May 2018 and, as of 30 April 2020, reported enrollment of >348,000 participants, of which 271,000 had completed initial steps for participation (https://www.researchallofus.org/data-snapshots/), although it is not clear when the program will be fully operational. Despite its large and diverse target sample, the All of Us program has significant limitations relative to the GoM CHOS, including lack of a statistically derived sampling plan, which restricts its utility in epidemiological contexts (46). Strong points are its size and national scope, emphases on enrolling underrepresented and minority participants, and plans for sharing data, software, and other program resources. Also, the All of Us questionnaires and protocols are similar to those used successfully in the BRFSS, NHANES, and NHIS surveys and have been validated in pilot studies, making them attractive as potential templates for the new cohort studies proposed here.

The three inner rings (Gold—Large Cohort; Yellow—Small Cohort; and White—Disaster-Specific Cohorts) would be new longitudinal cohort studies specifically designed for the GoM CHOS. Workshop 1 participants identified new cohort studies as essential elements of a GoM CHOS, and this finding is affirmed by recommendations from the literature (19, 37, 47, 48). This approach will enable a two-pronged sampling strategy: (1) a *prospective* approach that ensures that data are collected across the Gulf in anticipation of future disasters and (2) a *responsive* component consisting of disaster-specific cohort(s) that will be established immediately after a disaster. As envisioned, the new cohort studies will be nested, with the Small Cohort being a more intensively sampled subset of the Large Cohort, and the Disaster-Specific Cohort(s) drawing participants from the Large and Small Cohorts to the degree possible based on the location and time and geographic scales of a specific disaster. Participant recruitment and data collection methods will be tailored to ensure comparability and interoperability of data among all cohorts.

The Large GoM Cohort study (gold ring) is the largest of the new longitudinal cohorts developed specifically for the GoM. Its design was guided in part by other successful cohort studies, such as the All of Us, CARDIA, Dunedin, Framingham, MacArthur, MIDUS, and Wisconsin studies (1). As proposed, it will contain proportional representation of participants from coastal areas in all five states. The Large Cohort will include sociodemographic and self-reported mental and physical health information similar to that collected in the backbone studies. In addition, clinically relevant data will be collected via clinical visits, mobile monitoring, telemedicine methods, and remote sensing. Some community health metrics also will be included for all cohorts, as described below.

The yellow ring represents the Small GoM Cohort study, conceived as a subset of the Large Cohort (but still Gulf-wide) to provide more detailed health data. Small Cohort participants could be chosen based solely on willingness to provide more detailed health information, or to represent specific vulnerable populations of the Gulf, or there could be multiple small cohorts, each representing different geographic areas or serving initially as demonstration projects. Targeted sampling in the Large or Small Cohorts could facilitate the construction of other studies for more intensive sampling for different purposes (49, 50).

The inner white circle, the Disaster-Specific Cohort, is expected to be a considerably smaller cohort, established rapidly following a specific disaster and would likely consist of more exposed participants. However, if one considers a pandemic as a disaster, then the Disaster-Specific Cohort(s) would likely have to be larger and encompass a much greater geographic area than contemplated in the current design. Leaving this example aside for the moment, the white circle will be a further nested collection where participants are recruited from the Small Cohort as possible. Recruitment from the Large Cohort or of new participants may be necessary depending on the disaster and its characteristics. Creation of any disaster-specific cohort would be coordinated with local and state public health and emergency response officials, the CHOS community and scientific advisory committees, and other organizations and officials as appropriate.

### Design Options for Cohort Studies

#### Sampling Area

**Figure 2:**
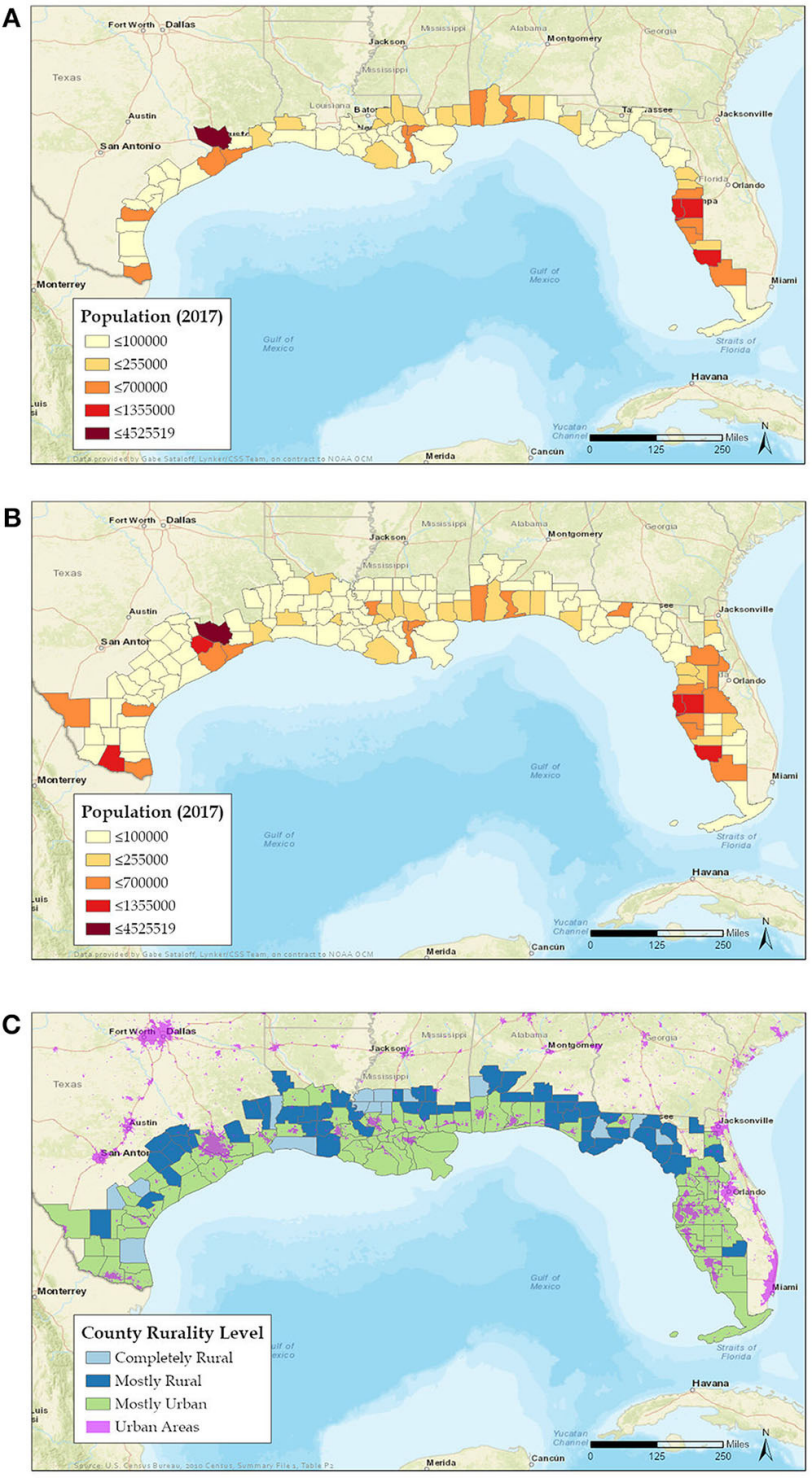
Coastal shoreline **(A)** and shoreline plus watershed **(B)** counties in the Gulf of Mexico region [adapted from (51), courtesy of G. Sataloff, NOAA]. Differences in color represent differences in relative population (53), with lighter shades indicating lower population and darker high. **(C)** Coastal shoreline and watershed counties showing relative levels of rural or urban characteristics (54).

Based on the geographic location of numerous previous disasters and the large size of the GoM region, we propose to limit the area covered by the GoM CHOS to the counties immediately along the Gulf coast. The National Oceanic and Atmospheric Administration (NOAA) categorizes coastal counties as *shoreline* or *watershed* (51, 52). Coastal *shoreline counties* have a coastline bordering the ocean or Great Lakes or contain areas identified by the Federal Emergency Management Agency (FEMA) as having high risk for tidal and/or storm surge flooding (Figure 2A) and are where the majority of economic production from coastal and marine-related natural resources is concentrated. Coastal *watershed counties* are those that lie immediately behind the shoreline counties and whose residents affect the coast but are generally less impacted by coastal disasters (Figure 2B). We recommend the 68-shoreline counties across the five GoM states be included as primary sampling targets for the GoM CHOS data collection (Figure 2A). This limits the spatial scope of the study area considerably, but still includes a substantial human population (~16,300,000 people) (53). In addition to being the areas typically most affected by hurricanes and major oil spills, people residing in the shoreline counties are more likely to be exposed to airborne oil spill chemicals (55) and red-tide HAB toxins (56). Many employees of coastal businesses may reside further inland and therefore outside the initial study area. These workers, their families and businesses, and social connections that support them are vulnerable to disaster events and should be considered for subsequent iterations of recruitment, with the coastal watershed counties offering a potential option for expanding the study area.

Although it will be impossible to state the actual sample size to be targeted until funding and other operational matters are decided, we did an example trial calculation [(1), p. 35]. This resulted in estimated targeted sample sizes of between 96 and 1,031 participants for each of the 68 coastal counties included in the framework, depending on the margin of error deemed acceptable at time of implementation. The total estimated sample for all counties was 6,528, 25,704, or 70,108 participants at 10, 5, and 3% confidence levels, respectively.

#### Population Sampling and Participant Recruitment

To the extent possible, the new cohorts should be created as random and representative samples that proportionally and adequately reflect health characteristics of the resident coastal populations in the five GoM states. The volunteer participant population should be composed of approximately equal numbers of adult men and women (18 yr and older), with no upper age limit, and children from age 3 to 18 with parental consent. Gender-specific information, including pregnancy status, will be obtained via questionnaires and examinations. Because health status and health-care access differ between rural and non-rural areas (57), and socioeconomic factors may increase vulnerability of rural residents (58), we propose a clustered, stratified random sampling design with urban and rural shoreline counties as the initial strata. Target counties and parishes would be stratified by either the Rurality Level (54) (Figure 2C) or the Center for Disease Control and Prevention (CDC)'s Urban-Rural Classification Scheme for Counties (59). Additional stratification at the U.S. Census Bureau tract or block group level or by ZIP code could occur within each selected county or parish using density of development derived from land cover data and/or population density.

Subpopulations likely to be most at risk from future natural and technological disasters can be defined by geographic location; social determinants of health as defined by the six social capitals (60); or other predisposing factors such as socioeconomic status and preexisting chronic health problems. One potential tool for identifying subpopulations is the Tapestry Segmentation dataset curated by Esri, which uses sociodemographic characteristics to classify U.S. neighborhoods into 67 distinct groupings from the county to the block group level. While designed for the small-area analysis of consumer markets, the groupings could either be used as-is or the cluster analysis techniques could be adapted using sociodemographic variables more specific to categorizing vulnerability (61).

Members of minority, underserved, and disadvantaged communities, including those with poorer health, typically yield the lowest response rates and/or have been poorly represented in epidemiological studies, potentially affecting the validity of study results (33, 62, 63). Purposeful oversampling in urban–suburban and rural areas may be necessary to ensure sufficient vulnerable or at-risk individuals are included. Also, minority communities are frequently located near industrial harbor and port facilities, with resulting disproportionate exposure to potentially health-damaging levels of petroleum hydrocarbons (64). Adding participants beyond those selected randomly or in the original sampling frame to meet project objectives of inclusiveness is a fairly common practice (33, 65–67). A variety of methods including adjusting for covariates of selection, inverse probability weighting, and sensitivity analyses can be employed to control for selection biases introduced by a targeted sampling design (67). In addition, direct recruitment via federally qualified health centers (FQHCs), involvement of trained community health workers (CHWs) to identify volunteers, and other means may be required.

Prior to recruitment of participants, the GoM CHOS should initiate a broad community engagement effort employing a community-based participatory research (CBPR) approach (68–72) and specifically including environmental justice communities. This should be a robust engagement and awareness campaign to inform the public about the CHOS, raise public awareness, provide information, and encourage participation utilizing public news media, GoM Sea Grant programs, healthcare providers, social media, community organizations, pharmacies, churches, grocery and convenience stores, and other willing outlets. The community engagement effort should begin at least 6 months before initiation of recruitment efforts and continue at a reduced level over the life of the CHOS.

Potential participants will be screened for enrollment in the Large or Small Cohort based on response to an initial mail inquiry about their willingness to participate and a limited amount of information that would allow provisional classification of vulnerability. Address-based sampling, which can be followed by acquisition of relevant electronic contact information, may be a better way to construct a participant sample frame than telephone or internet-based sampling (1, 73). Participants who are willing to provide more detailed information will be considered for enrollment in the Small Cohort. The initial mailing may be supplemented by personal contact to ensure recruitment of sufficient numbers of vulnerable people and those willing to provide additional information or samples as needed for the Small Cohort. Initial screening criteria will include but may not be limited to number of positive responses to vulnerability screening questions, gender identification, age, socioeconomic and educational status, race/ethnicity (e.g., African American, Hispanic, Vietnamese-American, other), and preexisting health conditions. Methods to bolster the number of participants in subpopulations could include additional mailings targeted to Census tracts known to be more heavily populated by people with lower socioeconomic status (SES) characteristics, or contacts with patrons of FQHCs (74). Another approach would be to train and deploy CHWs (75, 76) who could serve as study ambassadors in their communities, helping identify potential participants. Although utilization of CHWs in recruitment may introduce sampling bias, a wholly probabilistic sampling process may yield more respondents who have significantly greater social and health status than those of the overall community being studied (63, 65, 67). The non-response error introduced by a solely randomized sampling strategy is significant (77) and potentially greater than the biases introduced by recruiting individuals via a targeted approach (67).

### Data Collection

#### Primary Data

New data will be gathered through a mixture of (a) survey instruments; (b) clinical assessments of psychological and physiological health; (c) collection, processing, analysis, and banking of biospecimens and derived biomarkers; and (d) wearable health devices. Telemedicine approaches, the provision of medical services and information via electronic means, have proven useful for remote treatment of a variety of mental and physical ailments (78), collecting health data during a disaster (79), and providing health care during the current COVID-19 pandemic (80). All data collection and management activities will be conducted by appropriately trained and qualified personnel either in a clinical setting or via use of telemedicine methods.

We recognize that there is growing concern about whether or not the current practice of surveying by questionnaire is sustainable (81). At this time, we do not see a viable alternative to questionnaires for certain kinds of data, although such may develop in the future. Establishment and maintenance of a robust scientific advisory group that could assist the GoM CHOS to shift onto new technological platforms as they develop over time will be a crucial aspect of CHOS governance (see Discussion).

Institutional Review Board (IRB) protocols will be followed for all data collection. Upon receipt of signed informed consent, each participant will be provided detailed survey instruments by mail or electronic means depending on preference. Surveys may be filled out at home by the participant or with a trained interviewer. Involvement of interviewers can be important in situations where participants may have poor reading skills, lack facility in English, and/or have poor health and environmental literacy (72, 82, 83). Failure to provide for these circumstances may lead to elevated levels of anxiety and non-participation or non-compliance later in the study. However, in the presence of an interviewer, people may tend to offer responses that are considered socially desirable (84, 85). Unfortunately, no one surveying mode is generally accepted for all circumstances (86). A final decision as to how personally provided information (PPI) will be collected likely will be made when the program is implemented. For the present, we suggest that in most cases the initial data questionnaires be filled out by the individual participants, with the more expansive interviewer approach reserved for the Disaster-Specific Cohort(s) or for special language/literacy situations, possibly with assistance of CHWs. Regardless of whether interviewers are involved or not, all questionnaires, informed consent, and other significant program documents should be provided in Spanish and Vietnamese, in addition to English.

A suggested list of data and samples (Table 1) is presented as a starting point for implementation decisions. This list was developed based on expert input and literature review and should be sufficient for tracking health characteristics of the GoM coastal populations. Questions for the Augmented BRFSS, if implemented, should be prepared by Health Departments in the five Gulf States, in collaboration with the CDC. PPI will be elicited from questionnaires similar to and likely derived from the ongoing data domains included here (All of Us, BRFSS, NHANES, NHIS) and will include personal and some community information. Similarity to established survey instruments will ensure comparability of CHOS data to national averages.

**Table 1:** Types of data proposed for collection in Gulf of Mexico Community Health Observing System cohort studies.

**PPI from questionnaires**		**PPI from questionnaires**
Demographic information, including ethnicity, sex/gender identity, marital/partner status, children, residential history Socioeconomic information, including ability to deal with minor financial emergencies General health status Personal health history, including chronic, and major diseases Family health history, including chronic and major diseases, and occupational history Life history and behavioral factors, including alcohol, tobacco, and illicit drug use, nutrition, exercise, sleep Health care access and services utilization		Prescribed medications Previous disaster/trauma experiences including in childhood Residence and adequacy of housing Known or suspected exposure to toxic or infectious substances or organisms Social, religious, tribal, community attachments, and memberships Marginalization and discrimination (political, racism, ethnic, ageism, economic) Feeling of security or insecurity in home and neighborhood Level of trust in government/societal structures
**Mental health measures**	**Physical health measures**	**Biospecimens**
Anxiety: GAD-7 Depression: PHQ-8 or 9 PTSD/PTSS: PTSD Civilian Resilience: CD-RISC-10 (Connor-Davidson Scale) Alcohol abuse: AUDIT-C Religiosity: RQ-12 General self-efficacy scale (GSES) Social capital (adapted from Loneliness scale (ULS-8) Sense of control scale Cognitive function (IQ or other)	Systolic & diastolic BP Pulse (heart) rate Height & weight Waist–hip ratio Body mass index Lung function (FEV1/FEVC) Cardiovascular fitness Gum health Balance Ambulatory fitness (ability to rise, stand, walk)	Blood Plasma Serum Saliva Urine Hair DNA, mtDNA, telomere length (buccal swab) Nails (finger & toe) Stool Breath Umbilical cord blood (when available)

*All but PPI will be obtained in clinical settings. See Sandifer et al. (1) for reference citations*.

Collection of mental and physical health data (Table 1) will be done by qualified personnel following established biomedical protocols such as those detailed in the NIH GuLF study (87) and the All of Us program (45). Any measurement values indicative of a near-term health problem will be referred for evaluation, and, if deemed prudent, the participant will be recommended for an appropriate clinical examination. All data will be entered into a computer database and an individual's data will be made available to that participant.

For mental health, screenings will target depression, anxiety, PTSD, and others that are significantly associated with disasters (Table 1). Additional psychological indicators that measure social and interpersonal support, coping mechanisms, purpose in life, satisfaction with life, quality of life, domestic conflict, cognitive impairment, and experiences of specific disasters such as oil spills, hurricanes, and floods may also be useful (1).

Proposed physical health measurements (Table 1) are based on importance relative to disasters, use in other longitudinal studies, and relative ease of collection. Biological specimens will be collected from willing adult participants (18 yr and older) and, if possible, for young children with parental consent. The biospecimens identified, other than breath, are collected routinely in longitudinal studies and provide indicators of stress, frailty, health status, and exposures to potentially harmful substances (1). Because of the expense involved in biospecimen collection, storage, and analysis, it may be necessary to restrict most biospecimen collections to the Small Cohort and any Disaster Specific Cohort. Biospecimens will be aliquoted for both near-term analysis and storage for later use. Biobanking procedures will be based on established protocols [e.g., (45, 87)].

Choosing biomarkers is complicated by intended usages, storage and analytical costs, and stability in storage. Recommendations about biospecimen collection, and biomarkers are based on current analytical capabilities and are expected to change somewhat with future improvements in collection, analysis, and use. Many of the biomarkers recommended here (Table 2) have been widely used for a variety of clinical and research purposes, including health and exposure assessments and for calculating allostatic load (AL) (88). AL conceptually refers to “the price the body pays for being forced to adapt to adverse psychological or psychosocial or physical situations” (89). Development of AL in an individual can be thought of as the cumulative dysregulation of physiological mediators of adaptation over time. While no formulation of AL suitable for broad use is currently available, its potential for dynamic operationalization is the subject of a paper currently in preparation. Having a suite of biomarkers will support health assessments and development of AL and other health evaluation tools. Prior to implementation of the GoM CHOS, the list in Table 2 and all other health parameters proposed should be subjected to review by a panel of experts convened specifically for that purpose. Also, a carefully designed and managed process for reviewing requests for use of stored biological materials should be an integral part of the system implementation plan and should allow for input of supplementary resources from research interests outside of the CHOS, thus expanding uses of the observing system's resources. This will likely require a data sharing agreement.

**Table 2:** Biomarkers that have been used or suggested for use in assessing allostatic load and health status in longitudinal or other studies or recommended during an expert workshop.

**Neuroendocrine**	**Cardiovascular & Respiratory**
**Cortisol (diurnal, salivary, or urinary) ** Cortisone **Dehydroepiandrosterone (DHEA-S)** **Insulin-like growth factor (ILGF) ** **Norepinephrine ** **Epinephrine** Dopamine Aldosterone	**Systolic blood pressure** **Diastolic blood pressure** Mean arterial pressure **Heart rate (HR)** **Peak respiratory flow (FEV1)** Cardiorespiratory fitness Gum health Anti-hypertensive medication Glucose medication
**Immune**	**Anthropomorphic**
White blood cell count **Interleukin-6 (Il-6)** Tumor necrosis factor α **C-reactive protein** **Fibrinogen** Leukocyte telomere length Immunoglobulin E (IgE) Cytomegalovirus (IgC)	**Waist–hip ratio** Height Weight Waist–height ratio **Body mass index (BMI)** Facial age Underweight (%)
**Metabolic**	**Psychological/Cognitive**
Total cholesterol (TC) **High-density lipoprotein (HDL) ** **Low density lipoprotein (LDL)** Lipoprotein **TC:HDL ratio** **Triglycerides** **Glucose** **Insulin** Albumin **Glycosylated hemoglobin (HbA1c)** Creatinine (creatinine clearance) Homocysteine Urea nitrogen Alkaline phosphatase Apolipoprotein A, B100 ratio Liver enzymes mtDNA Inflammation marker	IQ test Sense of control Sleep issues Impairment of function Feeling unsafe in neighborhood Lack of neighborhood cohesion Financial strain Social isolation Loneliness Relationship conflict Discrimination Work stress Caregiving stress

*Items in bold type were most commonly used. See Sandifer et al. (1) for reference citations*.

Data collection intervals will be established at the time of program implementation. Annual updates of data are preferred, but program logistics may necessitate a different interval. Ideally, this will not be longer than 2–4 years. Add-on special studies or demonstration projects may require different sampling schedules. Disaster-Specific Cohort sampling will occur outside of the scheduled collection intervals as needed following disaster events, but should not supersede scheduled collections.

In addition to health data, monitoring for exposures to contaminants, toxins, organisms, and conditions associated with disasters should be an important component of the GoM CHOS (Table 3). Exposures can involve inhalation, contact, ingestion, and emotional pathways. Collection of exposure information should include questionnaires, monitoring of ambient levels of exposures of concern via existing databases and remote sensing, and analyses of urine, blood, and other biospecimens as necessary to assess body burdens of selected contaminants, toxins, or microorganisms of concern.

**Table 3:** Recommended list of environmental exposure information to be collected from questionnaires, analyses of biospecimens, and/or analyses of samples from homes, workplaces, or the environment of a particular disaster and included in cohort studies.

Particulates (PM_2.5_ and nanoparticles)
Air temperature extremes (hot and cold)
Unclean/contaminated drinking or recreational water
Oil and its components and other chemicals^*^
Contaminated or spoiled food
Pesticides
Harmful bacteria and viruses
Harmful algal blooms/toxins
Mold
Overexposure to sunlight
Radioactivity
High levels of psychological and physiological stress

(The CDC Environmental Public Health Tracking Network includes a much more extensive list of health effects and exposure indicators, including some community factors included elsewhere in this paper; see https://ephtracking.cdc.gov/showIndicatorPages).

* *For oil and its components, there are concerns with potential for polycyclic aromatic hydrocarbons (PAH) to contaminate seafood. However, the list of PAHs typically measured needs to be updated (90) as do lists of other chemicals of environmental concern related to human health*.

We anticipate that some cohort participants, particularly in the Small and Disaster-Specific Cohorts, will have or agree to be outfitted with wearable health devices (WHDs). Wearable or portable monitoring devices for health-related parameters including biomarkers, behaviors, and exposures are used widely for persons dealing with chronic illnesses such as CVD and diabetes, as well as by individuals to track personal health indicators. WHDs come in an almost bewildering variety of forms including wristbands; smartphones and apps; activity trackers and smartwatches; specialized monitors; high-tech patches; “smart” rings, clothing, glasses, contact lenses, ear monitors, arm bands, and jewelry; and even temporary tattoos that can be placed on or in the skin, as well as ingestible and implantable devices. For large-scale, long-term studies, WHDs should be non-invasive, inexpensive, simple, and relatively easy for both participants and data recorders to use, light weight and comfortable, relatively non-intrusive, robust and durable, have small power demands and/or long battery duration, provide accurate data, and include built-in security to protect data.

Smartphones are the most ubiquitous of passive sensors (91). Already, 96% of Americans own a cellphone of some type, 81% have a smartphone, and even 71% of those with incomes < $30,000/year have a smartphone (92). Smartphones should be a primary target for health monitoring due to their near ubiquity, familiarity, amount of useful data collected, and flexibility for addition of, or use in concert with, other monitoring devices or apps. Smartphones and apps, smartwatches, Fitbit® or similar types of activity and vital sign monitors have the ability to collect relatively accurate readings for blood pressure and heart rate in addition to providing location, relative position, periods of activity, ambient light, and humidity. These types of devices are being augmented rapidly with additional sensors, such as for respiration, blood O_2_ levels, sweat, and other parameters. Smartphones can also be used for “ecologic momentary assessment,” such as getting repeated responses to a few questions about a subject's immediate experiences of anxiety, pain, substance use, local environment, etc., over the course of a day or other time frame instead of relying on a single response that may be subject to recall bias (93).

Silicone wristbands appear to be the simplest, least expensive, and easiest to deploy WHD for capturing potential contact and inhalation exposure for a large number of potentially dangerous chemicals, including polyaromatic hydrocarbons (PAHs), polybrominated diphenyl ethers (PBDEs), polychlorinated biphenyls (PCBs), flame retardants, pesticides, pharmaceuticals, personal care products, dioxins, furans, some endocrine-disrupting chemicals, potential carcinogens, and other chemicals (94–96). Rapid progress also is being made in development of practical WHDs for cholesterol cortisol, glucose, and other parameters (1). Jiang et al. (97) demonstrated potential of a specially modified patch to detect thousands of exposures to microbes, insects, pets, wildlife, and chemicals. While many technical and other challenges to the broad use of WHDs remain (98), their potential to significantly enhance the specificity and breadth of data collected make them worthy of incorporation into longitudinal health studies.

### Secondary Data

Secondary sources can play important roles in informing primary data collection. Such sources include syndromic surveillance systems (SyS), electronic health records (EHRs), remote sensing (RS), social media, environmental data bases, volunteer health workers, and community data sources to the extent feasible and useful. EHRs and exposure data are expected to be most important for the Small and Disaster-Specific Cohorts. To guard against ecologic fallacy errors, supplemental data from community surveys, remote sensing, and similar secondary sources will not be assigned to individuals or used for epidemiological purposes. Instead, this information is expected to be used to help flesh out an individual's surrounding environmental context.

SyS is a public health early warning system that uses existing electronic health information, primarily from chief complaint forms, for early detection of disease outbreaks such as influenza-like illnesses (99). State SyS systems are coordinated through the CDC and, in recent years, have expanded to encompass hazards and disaster response (100–104). SyS could be more useful in disaster health impact monitoring if they were strengthened by increased sharing among the states, improving the base chief complaint data formats, and incorporating more mental health, hospital discharge diagnosis, Medicare and Medicaid, and death data (1).

EHRs collected and maintained by clinical entities are expected to be critical sources of health data, and GoM CHOS participants will be asked to authorize access to their EHRs. Willingness to do so will not be a requirement for participation but likely will be a major factor in selecting candidates for the Small and Disaster-Specific Cohorts. A well-established effort making broad use of EHRs is the OneFlorida Clinical Research Consortium [https://www.ctsi.ufl.edu/ctsa-consortium-projects/oneflorida/, (105)]. OneFlorida resources include an IRB, clinical informatics, community research facilitators, community engagement programs, participant recruitment services, information technology resources, research training and education, and a statewide biorepository capacity. The NIH All of Us program is also planning to make significant use of EHRs (46). Both the OneFlorida and All of Us programs have or are developing robust data management structures that could serve as models or possibly collaboration opportunities for the GoM CHOS program.

Remote sensing (RS) is useful for examining the spatial components of human–environment interactions (106), as well as for describing the physical impacts and dynamics of a disaster in near real-time. Air pollution, in particular fine particulate matter (PM _2.5_), is “the most consistent and robust predictor of mortality from cardiovascular, respiratory, and other diseases in studies of long-term exposure to air pollution” (107). Remotely sensed satellite data are being paired with ground-based data and modeled PM _2.5_ metrics to create composite exposure data sets (108). Validation for these technologies is ongoing, and the methods and uses are expected to grow rapidly. RS is also vital to monitoring and forecasting distribution of chemical contaminants such as oil spills (109) and occurrence of harmful algal blooms (110, 111) and harmful *Vibrio* bacteria (112). Additionally, a wealth of RS tools exists to characterize landscapes and built environments, including the Normalized Difference Vegetation Index (113–115) and NOAA's Coastal Change Analysis Program (116). The GoM CHOS will rely on water and air quality databases maintained by the US Environmental Protection Agency (EPA) and NOAA, the Public Health Exposome database (117), the National Centers for Environmental Information (NCEI) (https://www.ncei.noaa.gov), the Human Health Exposure Analysis Resource (HHEAR) supported by the National Institute of Environmental Health Sciences (NIEHS) (https://www.niehs.nih.gov/research/supported/exposure/hhear/index.cfm#), and others for information related to exposures to toxic or infectious agents. It should also track information from relevant animal health assessments such as studies on marine mammals exposed to oil spills and other contaminants (118, 119).

Additional supplemental data will be derived from the All of Us (if implemented as planned), BRFSS, NHANES, and NHIS surveys/studies, the American Community Survey (https://www.census.gov/programs-surveys/acs), General Social Survey (https://gss.norc.org/), Robert Wood Johnson Foundation county health rankings (https://www.countyhealthrankings.org/), the Southern Community Cohort Study (62, 74), CDC's National Environmental Public Health Tracking Network (https://ephtracking.cdc.gov/), Southern Poverty Law Center's Hate Map (https://www.splcenter.org/hate-map), National Flood Insurance Program's Community Rating System (https://www.fema.gov/national-flood-insurance-program-community-rating-system), and others as may be identified. Several of these may provide information of value for identifying community-level characteristics important to understanding disaster impacts. Such characteristics include economic vitality; industrialization; vulnerability of critical infrastructure; community trauma recovery history; community decision-making; poverty; chronic health disparities; effectiveness of government and other formal and informal institutions; community organizations and support structures; green and “blue” (water-associated) spaces; and parks and outdoor gathering places.

A key goal of the health observing system proposed is to allow segregation of data down to the individual level and aggregation up to the level of a household, neighborhood, or community. Coupling the original data collection of the system's cohort studies with ongoing, broad cross-sectional health surveys, and connecting them with secondary socioeconomic, community, and exposure information are expected to build an archive of longitudinal health histories that could be used to address cumulative effects of multiple traumatic events.

### Data Management

**Figure 3:**
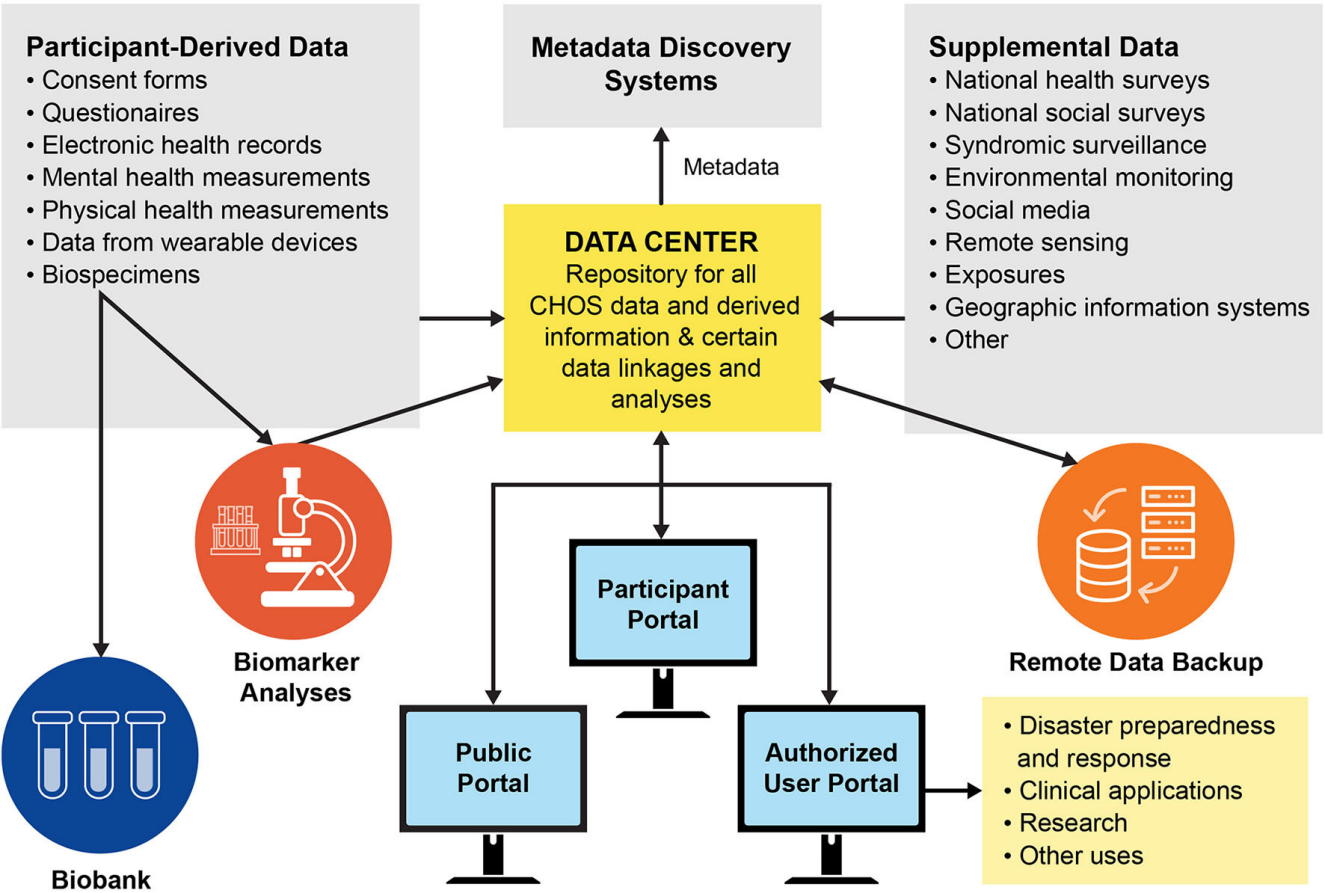
Data and specimen pathways for the Gulf of Mexico Community Health Observing System (biobank refers to long-term frozen storage of biological samples for later analysis).

We envision data management and biospecimen storage infrastructure for the GoM CHOS that would be managed by a third-party entity and similar to, and to the extent possible built upon, the All of Us and OneFlorida examples (Figure 3). In the All of Us program, a Raw Data Repository receives and stores all raw data in perpetuity in a secure system and facilitates safe transfer to a Curated Data Repository (CDR) and other systems. Only a small number of qualified personnel are allowed access to the raw data. The CDR provides organized data for access by users, but with robust protection of individual privacy. A Participant Technology System Center (PTSC) facilitates participant interaction with data and ensures access is recorded. All data are encrypted in the system and for transfers. Similarly, the OneFlorida Consortium has robust methods for handling, integrating, and analyzing the large amounts of data derived from EHRs. The All of Us and OneFlorida programs also have established secure biobanking protocols for long-term storage and access of biological specimens. For the GoM CHOS, access procedures for data users will be established at program implementation and will follow established security and use procedures. The ability to perform analyses at the individual level is vital to the calculation of numerous health metrics including AL. Analyses utilizing raw data collections will also allow the assigning of secondary data sets to individual participant files based on location.

The third-party data center will curate the primary and secondary data sets, accept new data sets, and ensure that all data are routinely backed up. ISO-standard metadata will be developed for each dataset generated. One option for making the metadata more discoverable is to submit them to NOAA's National Marine Fisheries Service InPort system (120). Additional options to improve data discovery should be explored at program implementation.

### Participant Retention

Participant retention is a problem in long-term studies due to death, loss of interest, change of economic or health status, social pressures, moving to another location, and other factors (121, 122). We propose to implement retention efforts similar to those used in previous successful studies (33, 66, 87, 123). As noted by Sandler et al. (87): “A key to high response rates and long-term participation is not to simply contact participants when data are needed but rather to maintain contact in small ways and provide useful information including study results back to participants on a regular basis.” Here, we propose that participants will receive information updates quarterly and a participant newsletter at least annually along with a request for information updates and intent to remain in the system. The periodic newsletter of the Wisconsin Longitudinal Study (https://www.wisls.info/) is an excellent example of a participant communication tool. A participant web portal also will be provided so that participants can easily access their information, update addresses, or other status information and ask questions, with support provided by a GoM CHOS staff participant liaison. Participants without regular electronic access will receive reports and other materials by mail. On occasion, participants will be polled to solicit opinions about their perceived value of the system and any criticisms or suggestions for improvements. Participants will be contacted ~6 months in advance of planned sampling intervals to confirm appointment times and other matters, and again at 3 and 1 month, and the week beforehand. Mobile phones, email addresses, emergency or family contact information, social media, and community organizations will be used as available and appropriate to attempt to contact and follow-up with any participants with whom contact has been lost or who move out of the study region. CHWs can also facilitate tracking of individuals, and subgroups with low-propensity for response can receive extra attention to enhance participation and retention (121, 124). As needed, new participants will be added to replace those lost via attrition.

In addition to the participant portal, the GoM CHOS should incorporate a public-facing web portal that will provide summary information on the program, semi-annual or annual updates on findings, and mechanisms for access to data by qualified individuals and organizations, which will specifically include State, County, and local Health Departments across the Gulf.

### Governance

Implementation of the GoM CHOS will require a single sponsor or more likely a consortium of several active institutional partners to oversee and implement the system and provide or raise the substantial amount of start-up and continuing funding needed to support dedicated staff and operations. The consortium or other management entity also should be responsible for regular reviews and assessments, financial and technical audits, and establishment of scientific and community advisory bodies to help guide the program. Although focused primarily on clinical work, the OneFlorida Consortium is an example of a highly successful program that might serve as a model for organizing the GoM CHOS or even as a potential partner. The OneFlorida governance structure includes representatives from all partner organizations, an IRB, and a scientific advisory board. Although the objectives of the GoM CHOS differ from those of the OneFlorida Consortium, there may be important opportunities for learning, partnering, sharing of information and experience, and adaptation of organizational approaches that could benefit the CHOS substantially. A GoM CHOS consortium could be organized through a partnership among entities already working in the GoM, such as the National Academies of Science, Engineering, and Medicine (NASEM) Gulf Research Program, the five Gulf State Health Departments, the NIEHS, and other Federal agencies. Private-sector and non-governmental organizations such as large health-oriented philanthropic foundations and major industries that have substantial work forces in the region (e.g., the petrochemical, tourism, and fisheries industries in the Gulf, others in different regions) could also be included. Some other governance options are explored briefly by Sandifer et al. (1).

## Discussion

The GoM CHOS is designed to collect, curate, and disseminate high-quality health-related data and biospecimens from thousands of GoM residents, with special attention to the most vulnerable and at risk. Data and information products are intended for use to enhance understanding of health effects of disasters, improve capacity to address immediate and long-term disaster health impacts, aid in directing health services to those most in need, and increase individual and community resilience (1). Primary audiences are expected to be public health personnel, emergency managers and responders, clinical and academic researchers/practitioners, and governmental agencies at all levels. Secondary users likely will include community leaders, planners, and organizations; natural resource managers; chambers of commerce, business associations, and private businesses; charitable and other non-governmental organizations; tribes and indigenous people; and community members.

Any program of the scope proposed here has both risks and benefits for participants. Based on experience elsewhere (45, 87), overall, risks appear minimal, and limited primarily to possible data breaches, uneasiness in providing certain kinds of information, and fear, discomfort, or minor risks associated with blood draws or other biospecimen collection. Programmatic risks include lapses in program comprehensiveness, continuity, or data security due to interruption in funding; management failures; lack of clearly defined, standardized, and enforced protocols; and poorly developed dispute resolution processes. Benefits are expected to include a small financial or other incentive, potential for identification of previously unrecognized health problems, regular medical check-ups, personal genetic information, being better prepared to deal with health impacts of future disasters, detailed personal health information for use in insurance or claims purposes, knowledge of personal contribution to improve disaster public health responses in their own region, and ultimately strengthened community resilience. An additional benefit will be the development of preventive public health strategies that alert the public in order to prevent or minimize exposure to disaster-associated health hazards.

Development of a practical, large-scale health observing system requires reliance on proven technologies to ensure reliability and cost-effectiveness of data gathered. At the same time, long-term success of a structured surveillance platform necessitates incorporation of new methods and technologies as their capabilities are verified. Such improvements are likely to occur in the rapidly evolving fields of WHDs, diagnostic tests, improved assessments of AL, genetic “fingeprinting” and integrated personal omics profiling (http://snyderlab.stanford.edu/iPOP.html) and digital health. Since health histories are of primary interest in longitudinal cohort studies, more personal, and less technology-based approaches such as nuanced, individual visual health histories (e.g., the Pictal Health system, https://www.pictalhealth.com/) also could be considered for inclusion. We recommend that, once necessary commitments to establish a GoM CHOS are made, an expert team be convened to develop final study design and implementation plans, including health data to be collected, population sample sizes, and other operational considerations.

As we were nearing completion of this study, the COVID-19 pandemic caused by the SARS-CoV-2 virus engulfed the world, infecting millions, killing hundreds of thousands, and catastrophically impacting the U.S. and global economies. More than any of the other kinds of disasters contemplated here, this pandemic has drawn attention to the urgent need for comprehensive, accurate, and rapidly responsive health surveillance at local, regional, national, and global scales (38, 125–127). Like other disasters, long-term health effects of the pandemic are expected to include serious mental health and stress-associated impacts (25–28). These will be exacerbated by the observed higher levels of COVID-19-related serious illness and mortality among the elderly and others with underlying health problems, people living or working in any type of close proximity or communal arrangements such as nursing homes, military installations, or prisons (126) or in areas with high levels of air pollution (128); health-care workers and others employed in occupations where social distancing is not possible; and people of color where impacts are likely amplified by long-term health disparities (129–131). In addition, men appear to suffer more serious illness and possibly higher mortality rates, in part perhaps due to sex differences in angiotensin-converting enzyme 2 (ACE2) receptors, relatively higher contribution of preexisting diseases (i.e., CVD, hypertension, diabetes, and chronic lung disease), higher risk behaviors (i.e., smoking and alcohol use), and occupational exposure (130, 132–137). More recently, there has been a rapid rise of cases of Multisystem Inflammatory Syndrome in Children associated with COVID-19 (138). Another complication may be the magnified stress and other health issues likely to be associated with managing stay-at-home, social distancing, and business closure directives related to this or future pandemics while simultaneously responding to mandatory evacuations necessitated by other, concurrent disaster events such as hurricanes, floods, and wildfires, as has recently occurred in the U.S. and elsewhere.

The need for broad-scale, regular sampling for COVID-19 and other emerging diseases in the U.S. and globally is real. The urgency is even more apparent when one considers the likelihood of continued problems with COVID-19, perhaps for 18–24 months (139) or longer, and the probability of more emerging pandemic diseases in the near future, with the U.S. as a likely major transmission node (140). Another reason for a robust surveillance system that can detect excess morbidity and mortality is the likelihood of corresponding spikes in the numbers of indirect illnesses and deaths attributable to disrupted health systems, delayed primary and preventive medical care, and forestalled treatment of complex medical conditions such as cancer or renal disease. These indirect consequences will find expression in excess mortality rates, similar to the excess deaths reported in events such as Hurricanes Katrina and Maria (141–143). The duration and breadth of social and economic disruptions associated with the global pandemic may extend the period during which such excess mortality and morbidity is observed. As the COVID-19 pandemic highlights, it is in the U.S.'s self-interest to protect not only its own residents but also the global population by taking all appropriate measures to prevent and mitigate future pandemics.

While many responsibilities appropriately belong in the public sector, much could be gained by developing and supporting robust public–academic–private partnerships that harness the powers, public health, and funding capacities of government; the cutting-edge research and analytical capabilities of academia; and the technological strengths and nimbleness of private businesses to address future health threats including pandemics. Examples abound of recent actions along these lines, such as Canada's and California's use of a private company, Blue Dot (https://bluedot.global/), to help track outbreaks, and enlistment of the private sector for rapid production of personal protective equipment and other biomedical necessities in the U.S. Israel even applied its military and intelligence-gathering infrastructure to enhance COVID-19 tracking (https://www.bbc.com/news/world-middle-east-52579475), although that approach raised concerns about personal privacy and potential for government overreach. Overall, however, these types of responses have appeared primarily as *ad hoc*, off-the-cuff reactions to the current COVID-19 threat, without consideration of long-term plans, needs, and consequences. This kind of approach is wholly insufficient for the future. One immediate need is for the Federal government to pre-certify a wide range of public (e.g., State Health Departments), academic, and private sector biomedical and clinical laboratories and require data sharing and inter-laboratory validation to ensure that adequate, accurate, rapid, and dependable sample analysis capacity is online prior to any future pandemics. It is equally important for all involved to speak with one voice, based on the available data.

Implementing regional and nationwide systems modeled on the GoM CHOS could be a momentous step toward meeting this need, especially if coupled to major academic (e.g., OneFlorida), business (e.g., Blue Dot), and local surveillance capacities (144). In particular, longitudinal cohort studies of the types described here that encompass large, well-characterized, and representative population samples could be expanded as needed to incorporate testing for COVID-19 and other infectious organisms at regular intervals. Such testing might focus on up-to-date technology based on minimally invasive, often self-administered, sampling [e.g., tests of saliva (145, 146), nasopharyngeal swabs (144), dried blood spots (147–149), urine (150, 151), and perhaps smartphone apps or other mobile monitoring health devices] to complement existing disease surveillance efforts. Broad scale surveillance for SARS-CoV-2 in wastewater is also possible, as already demonstrated in multiple localities (152–154). The observing system could adapt rapidly as new technologies for identifying SARS-CoV-2 and other emerging infectious agents are validated and become available and provide significant and timely information for infection tracing efforts. Equally important, longitudinal cohorts would allow tracking of pandemic health effects over a relatively long time period, including incidence and prevalence of asymptomatic disease; pediatric disease (https://www.nih.gov/news-events/news-releases/study-determine-incidence-novel-coronavirus-infection-us-children-begins); possible recurrence or reinfection; and interaction with other health factors such as underlying chronic disease, psychosocial impacts, and acute, sustained, and cumulative stress. Some ongoing longitudinal studies are already being adapted for such purposes (155). Data from longitudinal cohort studies could be integrated with information from one or more national cross-sectional studies that enrolled a new, randomly selected participant wave each year. Combined with rapid testing, tracking and modeling supplied via public, academic, and private sector entities, such an approach could deliver short- and long-term data to public health officials, clinicians, researchers, and the public to support robust interventions to future pandemics and other environmental and economic catastrophes.

## Data Availability Statement

This is a derivative work. As such no new data were generated and data were not analyzed. Many previous studies were reviewed and a new health observing (surveillance) system was designed focused on effects disasters have on human health, with a geographic focus on the Gulf of Mexico region. Literature review and expert workshops informed the design of the new system. Detailed results of the review and design are summarized in a supporting Technical Report (1).

## Author Contributions

PS and BS were responsible for concept formulation, funding, workshops, and overall content. PS had primary writing, literature review, and project management responsibilities, with yeoman support from LK and RM. LK contributed significantly to writing, review, and workshop planning. ML, TC, BM, TS, and RY were members of the project Steering Committee. DA, TC, LE, YH, BM, GM, LP, AP, TS, ES, and DY were invited workshop speakers. PS, BS, LK, ML, RM, DA, TC, KE, JF, MF, CH, EH, LH, BM, GM, RO, LP, MP, DP, GS, HS-G, FY, and DY reviewed and/or edited manuscript drafts. All authors except MP and FY participated in the expert workshop and all authors contributed ideas, read, and approved the submitted version.

## Conflict of Interest

AW was employed by the SEA Consulting Group and MF was employed by the Rand Corporation, a non-profit research organization. All authors declare that the research was conducted in the absence of any commercial or financial relationships that could be construed as a potential conflict of interest.
